# Diagnostic Utility of Podoplanin Immunohistochemistry Combined with the NanoSuit-Correlative Light and Electron Microscopy Method for Thoracic Malignant Tumors

**DOI:** 10.3390/diagnostics15101298

**Published:** 2025-05-21

**Authors:** Shin-ya Katsuragi, Yuri Sakano, Isao Ohta, Hisami Kato, Rei Ishikawa, Hirofumi Watanabe, Ryosuke Miyazaki, Katsuhiro Yoshimura, Hidetaka Yamada, Yasuhiro Sakai, Yusuke Inoue, Yusuke Takanashi, Keigo Sekihara, Kazuhito Funai, Yoshiro Otsuki, Hideya Kawasaki, Kazuya Shinmura

**Affiliations:** 1 Department of Tumor Pathology, Hamamatsu University School of Medicine, Hamamatsu 431-3192, Japan; skpath@hama-med.ac.jp (S.-y.K.); hisami@hama-med.ac.jp (H.K.); reipatho@hama-med.ac.jp (R.I.); d19033@hama-med.ac.jp (H.W.); 07486215@hama-med.ac.jp (R.M.); ky@hama-med.ac.jp (K.Y.); h-yamada@hama-med.ac.jp (H.Y.); ya-sakai@hama-med.ac.jp (Y.S.); 2 NanoSuit Research Laboratory, Division of Preeminent Bioimaging Research, Institute of Photonics Medicine, Hamamatsu University School of Medicine, Hamamatsu 431-3192, Japan; a20044@hama-med.ac.jp; 3 Advanced Research Facilities and Services, Division of Preeminent Research Supports, Institute of Photonics Medicine, Hamamatsu University School of Medicine, Hamamatsu 431-3192, Japan; ohtaisao@hama-med.ac.jp; 4 Second Division, Department of Internal Medicine, Hamamatsu University School of Medicine, Hamamatsu 431-3192, Japan; yinoue@hama-med.ac.jp; 5 Department of Surgery 1, Hamamatsu University School of Medicine, Hamamatsu 431-3192, Japan; nashimed1@gmail.com (Y.T.); sekihara@hama-med.ac.jp (K.S.); kfunai@hama-med.ac.jp (K.F.); 6 Department of Pathology, Seirei Hamamatsu General Hospital, Hamamatsu 430-8558, Japan; otsuki@sis.seirei.or.jp

**Keywords:** epithelioid malignant pleural mesothelioma (EMPM), lung adenocarcinoma (LAC), lung squamous cell carcinoma (LSCC), microvilli, NanoSuit-correlative light and electron microscopy (CLEM) method, non-small-cell lung carcinoma (NSCLC), podoplanin (PDPN), field emission–scanning electron microscope (FE-SEM)

## Abstract

**Background/Objectives:** Differentiating thoracic malignant tumors, such as epithelioid malignant pleural mesothelioma (EMPM) and non-small-cell lung carcinoma (NSCLC), primarily comprising lung adenocarcinoma (LAC) and lung squamous cell carcinoma (LSCC), remains a challenge in routine pathological diagnosis. This study aimed to evaluate whether podoplanin (PDPN) immunohistochemistry combined with scanning electron microscopy (SEM) using the NanoSuit-correlative light and electron microscopy (CLEM) methods could serve as a reliable tool for distinguishing these thoracic malignancies. **Methods/Results:** Initially, PDPN expression was assessed by immunohistochemical analysis in 11 EMPM, 100 LAC, and 23 LSCC cases. PDPN positivity was predominantly observed in the cell membrane and was significantly more frequent in EMPM (100%) than in LAC (2%; *p* < 0.0001) or LSCC (43.5%; *p* = 0.0018). Subsequently, field emission–SEM (FE-SEM) observations of PDPN-positive sites on immunohistochemical slides, conducted using the NanoSuit-CLEM method, revealed distinctive ultrastructural features. EMPM exhibited densely packed, elongated microvilli, whereas such structures were absent in LAC and LSCC. Furthermore, analysis of thick-cut sections (20 μm) demonstrated extensive microvilli coverage characteristic of EMPM. **Conclusions:** These findings suggest that the combined approach of PDPN immunohistochemistry and FE-SEM observation of PDPN-positive sites, using the NanoSuit-CLEM method, constitutes an effective diagnostic strategy for enhancing the accuracy of distinguishing EMPM from NSCLCs.

## 1. Introduction

Thoracic malignant tumors, including epithelioid malignant pleural mesothelioma (EMPM) and non-small cell lung carcinoma (NSCLC), primarily comprising lung adenocarcinoma (LAC) and lung squamous cell carcinoma (LSCC), present significant diagnostic challenges owing to their overlapping clinical and histopathological features in certain clinical contexts [1,2,3]. Lung cancer remains the leading cause of cancer-related mortality worldwide, and NSCLC accounts for approximately 85% of all cases [4,5,6,7]. Although relatively uncommon, EMPM is a highly aggressive malignancy that is primarily associated with asbestos exposure [1,4]. However, non-asbestos etiological factors, including exposure to naturally occurring mineral fibers such as erionite and previous therapeutic radiation, have been implicated in its pathogenesis [8]. EMPM is characterized by rapid disease progression, limited treatment options, and poor prognosis, underscoring the need for early detection and effective therapeutic strategies [1,2,3]. Accurate differentiation among these tumors is essential because their respective treatment strategies and prognoses differ considerably. Podoplanin (PDPN), a transmembrane glycoprotein, has emerged as a valuable biomarker for distinguishing EMPM from NSCLCs [9,10,11]. According to recent guidelines for the pathologic diagnosis of mesothelioma [9], PDPN is expressed in approximately 93% of epithelioid mesotheliomas, but in only approximately 3% of LAC cases. This differential expression permits employing PDPN immunohistochemistry for diagnostic pathology. However, previous studies have reported variations in PDPN positivity. Amatya et al. [11] observed a high positivity rate of 7% in LAC, whereas PDPN expression in LSCC ranged from 15 to 66% [12,13,14]. These findings indicated that PDPN immunohistochemistry alone may not reliably differentiate between EMPM and NSCLCs. Consequently, the current diagnostic consensus for EMPM relies on the use of multiple antibodies in immunohistochemistry, which increases diagnostic costs, demands more resources for slide preparation, and lengthens the time required for diagnosis.

Advancements in electron microscopy have significantly enhanced the ability to visualize biological specimens at nanometer-scale resolution [15]. However, conventional sample preparation techniques for scanning electron microscopy (SEM) involve chemical fixation, dehydration, and metal coating, which can introduce structural artifacts and compromise the hydration state of biological samples. These limitations hinder the accurate representation of native biological structures. To address these challenges, the NanoSuit method was developed as a biomimetic technology that enables high-resolution imaging of biological specimens, such as *Drosophila* larvae, using field emission–scanning electron microscopy (FE–SEM) without the need for conventional dehydration or fixation [16].

This technique involves the formation of an ultrathin polymer layer on the surface of a specimen to preserve its hydration state and structural integrity, thereby mitigating the key limitations associated with traditional SEM sample preparation methods. The NanoSuit approach has proven to be highly advantageous in biomedical and biological research because it facilitates the observation of hydrated biological specimens with minimal artifacts [17]. A key advantage of this method is its ability to suppress the charging effects caused by electron beam irradiation, while maintaining the natural morphology of biological samples [17]. This enables the high-resolution imaging of cells, tissues, and microorganisms with improved structural fidelity. Moreover, integrating the NanoSuit technique with correlative light and electron microscopy (CLEM) provides a powerful approach for correlating optical light microscopy with electron microscopy images, allowing comprehensive structural and compositional analyses of biomedical specimens [18].

The integration of NanoSuit technology with CLEM, referred to as NanoSuit-CLEM, enhances the precision of SEM imaging by incorporating immunohistochemical labeling techniques. The combination of osmium or gold chloride with 3,3′-diaminobenzidine (DAB) staining increases contrast in backscattered electron (BSE) mode, enabling selective visualization of specific cellular components [17,18]. This method is particularly beneficial for analyzing formalin-fixed paraffin-embedded (FFPE) tissue specimens and facilitating the precise correlation of pathological lesions between different imaging modalities. By combining light microscopy and SEM images, NanoSuit-CLEM can be a powerful tool for detailed histopathological analysis. One of the critical challenges in SEM-based pathology is the difficulty in correlating grayscale SEM images with light microscopy-based identification of pathological lesions. To address this issue, NanoSuit-CLEM incorporates fiducial marking techniques [18]. In this process, the target regions are first identified using light microscopy, and digital images are acquired. After removing the cover glass, a thin film of NanoSuit solution is applied to the specimen surface using a spin coater. An additional NanoSuit solution is placed around the target area to serve as a fiducial marker, improving positional accuracy during SEM observation. Unlike traditional CLEM approaches that require expensive custom sample holders and specialized imaging systems, this technique is cost-effective and minimizes artifacts. Furthermore, the NanoSuit membrane was removed after SEM imaging, allowing subsequent hematoxylin and eosin (H&E) staining for re-evaluation of the same tissue section. This feature offers the unique advantage of enabling sequential multimodal imaging, thereby enhancing the reliability of histopathological diagnoses. The NanoSuit-CLEM method has been used to observe various ultrastructures, including primary cilia in cancer cells, human papillomavirus in the mesopharynx, and lanthanum phosphate deposition in the gastrointestinal mucosa [19,20,21,22]. Its greatest advantage lies in its ability to perform SEM observations on FFPE specimens while correlating the findings with corresponding H&E-stained images or immunohistochemical images [18]. This versatility enables a wide range of applications and offers the potential for new discoveries.

In this study, we hypothesized that the ultrastructural differences in the immunohistochemically positive region for PDPN between EMPM and NSCLCs facilitate their differential diagnosis. To test this hypothesis, we performed SEM observations of PDPN-positive sites in these thoracic malignant tumors using the NanoSuit-CLEM method to develop a simple and practical approach for routine pathological diagnosis.

## 2. Materials and Methods

### 2.1. Case Collection

FFPE tissue blocks were obtained from 11 primary EMPM (mean age ± standard deviation: 59.2 ± 10.6 years; gender distribution: male: female = 9:2), 100 primary LAC (mean age ± standard deviation: 70.3 ± 7.65 years; gender distribution: male: female = 54:46; pathological tumor stage [pT]: pT1:pT2–4 = 60:40; pathological node stage [pN]: pN0:pN1–2 = 76:15), and 23 primary LSCC cases (mean age ± standard deviation: 71.9 ± 5.37 years; gender distribution: male: female = 20:3; pT: pT1:pT2–4 = 6:17; pN: pN0:pN1–2 = 19:3) at Hamamatsu University Hospital. Detailed case information is provided in Supplementary Tables S1–S3. For the pathological diagnosis of EMPM, immunohistochemical slides with several antibodies were used in addition to H&E-stained specimens (representative images are provided in Supplementary Figure S1). Thoracic malignant tumor samples were used for immunohistochemical analyses. The study protocol was reviewed and approved by the Institutional Review Board of the Hamamatsu University School of Medicine [approval numbers: 15-067 (17 July 2015) and 23-348 (27 February 2024)].

### 2.2. Immunohistochemical Analysis

Immunohistochemical staining was performed on the FFPE tissue sections using an automated system (Autostainer; DAKO, Carpinteria, CA, USA) to ensure standardized and reproducible staining conditions. Initially, FFPE sections were deparaffinized in xylene, rehydrated through a graded ethanol series, and rinsed in distilled water. As antigen retrieval was not required for the mouse anti-PDPN monoclonal antibody (clone D2-40, DAKO), the sections were directly treated with a hydrogen peroxide solution to block endogenous peroxidase activity, thereby minimizing nonspecific background staining. Subsequently, the sections were incubated with the primary antibody at a dilution of 1:200 under optimized conditions. Following primary antibody incubation, sections were rinsed with buffer and treated with a horseradish peroxidase-conjugated polymer detection system (Histofine Simple Stain MAX PO; Nichirei Biosciences, Tokyo, Japan) to facilitate signal amplification. For visualization, the sections were incubated with DAB as a chromogenic substrate, enabling the detection of immunoreactivity. After DAB development, the slides were counterstained with hematoxylin, dehydrated using graded ethanol and xylene, and coverslipped for microscopic examination. Positive and negative controls were included in each staining batch to ensure the specificity and reliability of the staining procedure. Immunostaining and H&E staining were performed using a Leica DMD108 digital microimaging system (Leica Microsystems, Wetzlar, Germany).

In this study, tumors were classified as PDPN-positive if more than 3% of the tumor cells were positive under conventional light microscopy. Tumors that did not meet this threshold were classified as PDPN-negative. Although 3% is a relatively low cut-off value, this threshold was adopted considering the limited nature of biopsy samples, which often contain only a small portion of the tumor. Even when the overall expression of the marker is low, there remains a possibility that a few positive cells may be present within the sampled area by chance. Therefore, this cut-off was established to account for such sampling variability. Although this specific cut-off is not established for PDPN, similarly low thresholds have occasionally been applied in the immunohistochemical evaluation of other biomarkers in the literature to accommodate sampling limitations and heterogeneous expression patterns [23,24,25].

### 2.3. FE-SEM Analysis of PDPN-Positive Site Using the NanoSuit-CLEM Method

**Figure 1:**
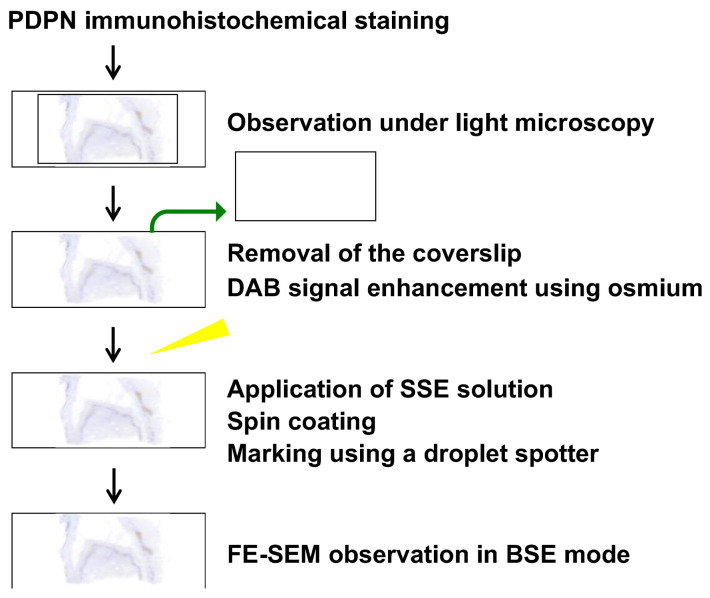
Schematic overview of the FE-SEM analysis of a PDPN-positive site using the NanoSuit-CLEM method. Detailed procedures are described in the main text.

Sections prepared for PDPN immunohistochemistry were also subjected to FE-SEM observations using the NanoSuit method [16,17,18,19]. A schematic overview of the procedure is presented in Figure 1. After acquiring the digital image data, a tissue section containing the region of interest was marked on both the front of the coverslip and the back of the microscope slide using a water- and organic solvent-resistant pen. The mark on the back of the slide served as a reference point for subsequent markings with the surface shield enhancer (SSE) solution. The diaphane and coverslip were subsequently removed from the slide. Following the enhancement of DAB signal using osmium, a diluted SSE solution was applied to the surface of the tissue section, covering the entire slide. The sample was left to stand for 1 min, after which excess SSE solution was removed via spin coating (2000 rpm, 15 s). SSE stock solution was prepared by dissolving sucrose, fructose, and sodium chloride in distilled water, followed by the addition of citric acid and sodium glutamate (pH 7.4). The resulting aqueous solution was then mixed with glycerin in a 1:2 ratio and subsequently diluted 20-fold for use. An on-demand droplet spotter (Hamamatsu Nanotechnology Co., Hamamatsu, Japan) was used to mark the designated pathological site precisely. This system can dispense minute volumes (pL–fL) of solution and is compatible with high-viscosity liquids such as SSE [18]. Upon the application of a pulse voltage between the liquid and substrate, a jet was ejected from the apex of the glass capillary to form a droplet. The SSE stock solution was applied to minute droplets at multiple precise locations using a computer-controlled droplet spotter system, targeting the black mark on the slide.

Following rehydration, the sections were directly introduced into the FE-SEM system (S-4800; HITACHI, Tokyo, Japan), where NanoSuits were formed in situ under electron beam irradiation. FE-SEM observations using the NanoSuit-CLEM method were conducted using a BSE detector equipped with an yttrium aluminum garnet (YAG) crystal (YAG-BSE) detector. This approach provides a stable, conductive, and artifact-free imaging environment, ensuring that high-resolution electron microscopy is suitable for detailed ultrastructural analyses.

### 2.4. Statistical Analysis

Statistical analyses were performed using Fisher’s exact test. GraphPad QuickCalcs (GraphPad Software Inc., San Diego, CA, USA) was used for the statistical analyses. *p*-values of less than 0.05 were considered statistically significant.

## 3. Results

### 3.1. Differences in PDPN Immunostaining Positivity Among Thoracic Malignant Tumors

**Figure 2:**
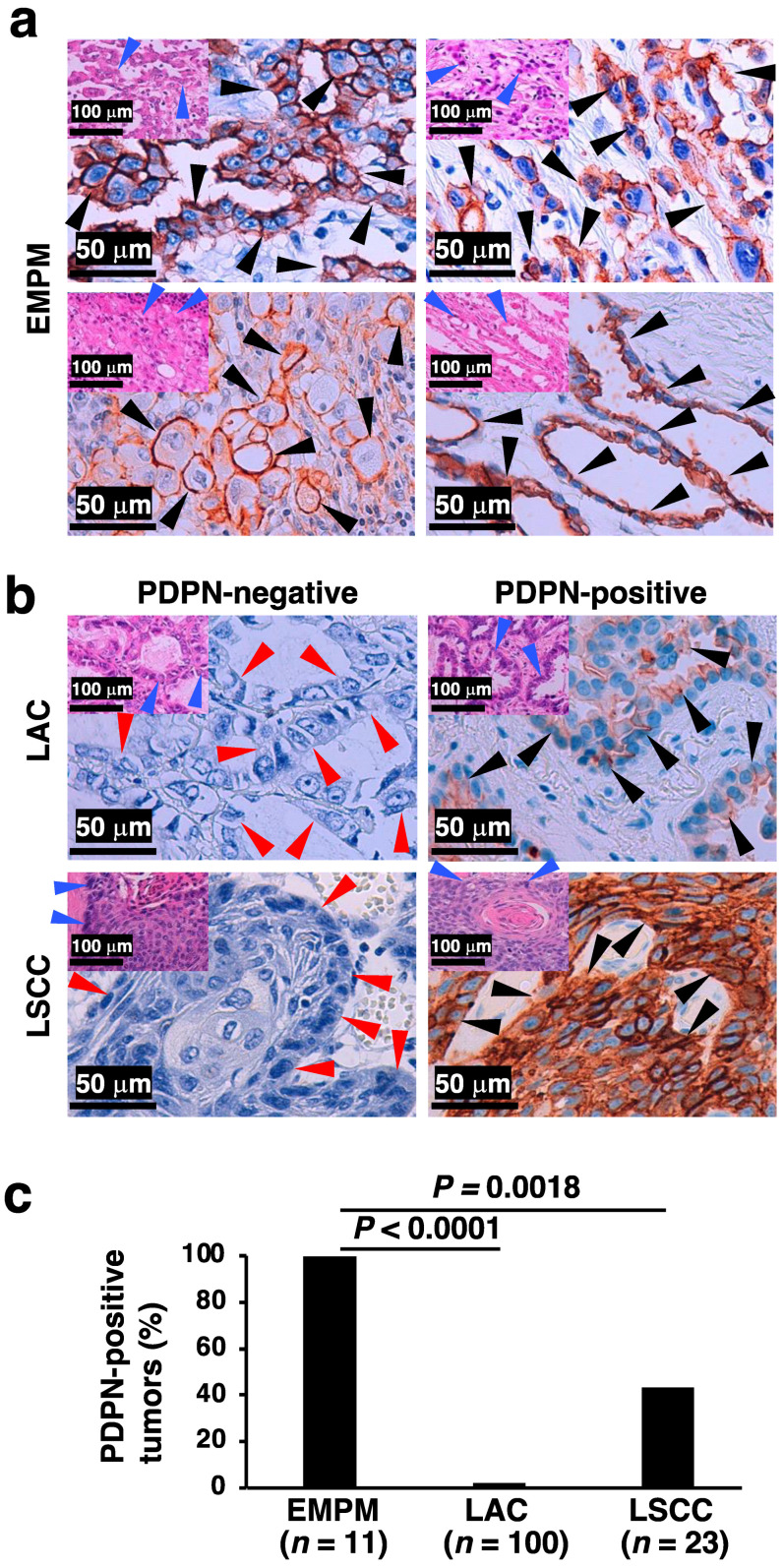
PDPN immunohistochemical status in thoracic malignant tumors. (**a**) Representative PDPN immunohistochemical staining images of four EMPM cases, demonstrating tubulopapillary (top left), trabecular (top right), deciduoid (bottom left), and acinar (bottom right) proliferation patterns. Insets in the upper left corner of each panel show corresponding H&E-stained images. (**b**) PDPN immunohistochemical staining images of two LAC and two LSCC cases. The left panels represent PDPN-negative cases, while the right panels represent PDPN-positive cases. Insets in the upper left corner of each panel show corresponding H&E-stained images. (**c**) Proportion of PDPN-positive tumors among EMPM, LAC, and LSCC cases. Statistical analysis was conducted using Fisher’s exact test. Black arrowheads indicate PDPN-positive tumor cells in immunohistochemically stained images, and red and blue arrowheads denote the architectural patterns of tumor cell proliferation in the immunohistochemical and H&E-stained images, respectively.

Immunostaining for PDPN is a well-established marker for differentiating EMPM from NSCLCs. In this study, we analyzed the PDPN immunostaining profiles of malignant thoracic tumors diagnosed at our institution, including EMPM (*n* = 11), LAC (*n* = 100), and LSCC (*n* = 23). Tumor cell positivity for PDPN immunoreactivity was significantly more frequent in EMPM (11/11, 100%) than in LAC (2/100, 2%; *p* < 0.0001) or LSCC (10/23, 43.5%; *p* = 0.0018) (Figure 2a,b: representative immunohistochemical results; Figure 2c: bar graph). No significant associations were identified between PDPN immunoreactivity and clinicopathological factors in LAC and LSCC, as detailed in Supplementary Tables S4 and S5, respectively. In the analyzed specimens, membranous PDPN staining was observed across all three tumor types; however, the proportion of stained areas differed between EMPM and NSCLCs. Specifically, EMPM showed a relatively diffuse staining pattern, whereas LAC and LSCC showed focal staining. These findings are consistent with those of a previous study [10]. Furthermore, as is widely recognized [26], positive PDPN signals were identified in the stromal lymphatic vessels of all three tumor types (Supplementary Figure S2).

Our observation of cell membrane-associated PDPN distribution, which is common across the three tumor types, along with the difference in PDPN positivity rates and areas between EMPM and NSCLCs, led us to hypothesize that structural differences at PDPN-positive sites may exist between these tumor types. Such structural variations could further enhance the diagnostic utility of PDPN immunostaining in distinguishing EMPM from NSCLCs.

### 3.2. FE-SEM Observation of PDPN-Positive Sites in Thoracic Malignant Tumors Using the NanoSuit-CLEM Method

**Figure 3:**
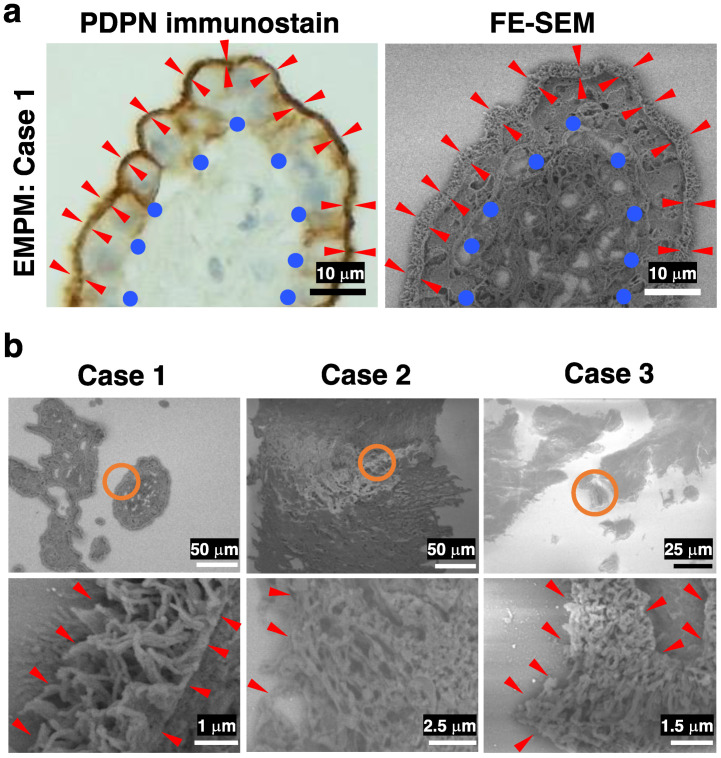
FE-SEM observation of immunohistochemical PDPN-positive sites in three cases of EMPM using the NanoSuit-CLEM method. (**a**) Representative paired images of PDPN immunohistochemical staining (left) and FE-SEM imaging (right) obtained using a YAG-BSE detector via the CLEM technique in Case 1 of EMPM. Briefly, PDPN-stained immunohistochemical slides—with coverslips removed—are treated with SSE solution following DAB enhancement with osmium. After region marking using a droplet spotter, the slides are directly introduced into the FE-SEM system, where NanoSuit formation is induced by electron beam irradiation. FE-SEM imaging is performed in the BSE mode. Red arrowheads indicate the PDPN-positive, exposed surface of mesothelial cells surrounding fibrovascular connective tissue, which is denoted by blue circles. (**b**) FE-SEM images showing PDPN-immunopositive sites in Cases 1–3 of EMPM. The regions enclosed by orange circles in the upper panels are shown at higher magnification in the lower panels. Red arrowheads highlight regions where the surface of tumor cells is exposed to the extracellular space.

We subsequently investigated the structural differences between EMPM and NSCLCs. For this, we used the NanoSuit-CLEM method for electron microscopic observation of membranous PDPN staining sites. DAB, commonly used in conventional immunohistochemical staining and optical microscopy, has an affinity for osmium, a component of electron microscopy. Consequently, the DAB deposition sites identified by optical microscopy correspond to regions of high electron reflection under an electron microscope. We performed PDPN immunostaining in EMPM and NSCLC samples, followed by osmium staining and FE-SEM observations using the NanoSuit-CLEM method. Paired images of PDPN immunostaining and FE-SEM with a YAG-BSE detector were successfully obtained (representative images are shown in Figure 3a; Case 1 of EMPM). Using this technique, high electron reflection sites in the YAG-BSE images, corresponding to the DAB deposition sites, were observed in the three EMPM cases (Cases 1–3) (Figure 3b). Under magnification, these regions revealed densely packed, thick, and elongated villous structures, indicating well-developed microvilli (Figure 3b, bottom row).

**Figure 4:**
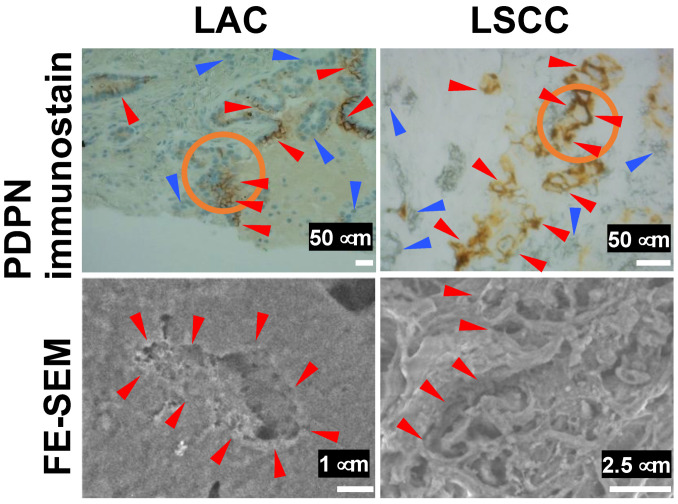
FE-SEM observation of immunohistochemical PDPN-positive sites in LAC and LSCC using the NanoSuit-CLEM method. The upper panels show PDPN immunohistochemical staining, while the lower panels present FE-SEM images captured using a YAG-BSE detector for a case of LAC and a case of LSCC. The areas enclosed by orange circles in the upper panels are presented as higher-magnification FE-SEM images in the corresponding lower panels. Red and blue arrowheads in the immunohistochemical images indicate PDPN-positive and PDPN-negative tumor cells, respectively. Red arrowheads in the FE-SEM images highlight regions where the surface of tumor cells is exposed to the extracellular space.

In contrast, a similar analysis of LAC and LSCC revealed poorly nourished microvilli in DAB deposition areas in both LAC and LSCC in the YAG-BSE mode (Figure 4). These findings indicate that PDPN immunostaining effectively highlights well-developed microvillus structures in EMPM, whereas such structures were not observed in LAC or LSCC. These findings suggest that PDPN immunohistochemistry is effective for distinguishing between EMPM and NSCLC. Moreover, when tumor cells are PDPN-positive, observing the corresponding site on the immunohistochemical slide using FE-SEM with the NanoSuit-CLEM method further enhances the accuracy of the differential diagnosis.

### 3.3. FE-SEM Using the NanoSuit-CLEM Method for the Detailed Characterization of Surface Structures in Thick-Cut Sections of EMPM

**Figure 5:**
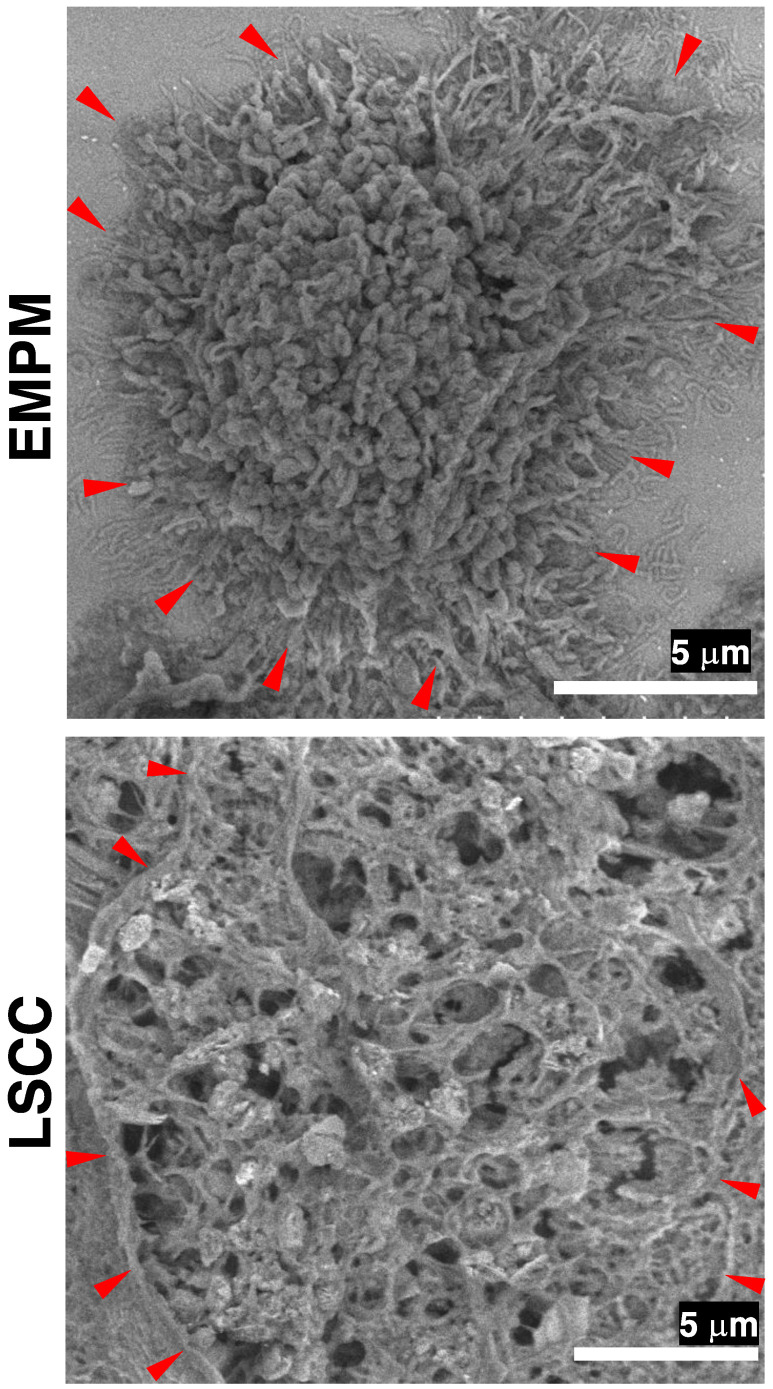
FE-SEM analysis of thick-cut section samples from EMPM and LSCC using the NanoSuit-CLEM method. FE-SEM images of a tumor cell from EMPM and a tumor cell from LSCC are obtained using a YAG-BSE detector. Red arrowheads indicate regions where the surface of tumor cells is exposed to the extracellular space.

To emphasize cell surface morphology rather than thin cross-sectional views, we prepared thick-cut sections (20 μm) of EMPM. Subsequent FE-SEM observations of PDPN-positive sites in the YAG-BSE mode, employing the NanoSuit-CLEM method, revealed that the entire surface of individual EMPM cells was covered with dense, elongated microvillar structures (Figure 5). In contrast, such microvillar structures were absent on the surface of tumor cells in thick-cut sections of LSCC, where the surface appeared rugged and lacked organized and elongated projections (Figure 5). These findings indicate that the NanoSuit-CLEM method, in conjunction with FE-SEM analysis of thick-cut sections, facilitates detailed characterization of the fine surface morphology of EMPM cells.

### 3.4. Proposed Workflow for Differential Diagnosis of Thoracic Malignant Tumors

**Figure 6:**
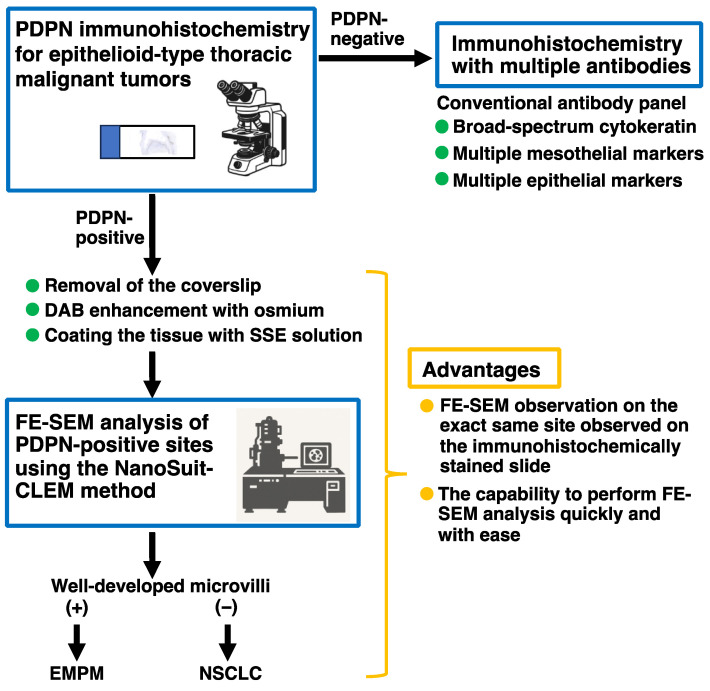
Proposed workflow for the differential diagnosis of thoracic malignant tumors using PDPN immunohistochemistry and subsequent FE-SEM analysis of PDPN-positive sites via the NanoSuit-CLEM method.

Based on our results, we prepared a workflow for the differential diagnosis of malignant thoracic tumors using PDPN immunohistochemistry and subsequent FE-SEM analysis of PDPN-positive sites using the NanoSuit-CLEM method (Figure 6). In this workflow, PDPN immunohistochemistry alone, rather than a multi-antibody panel, served as the initial diagnostic step for epithelioid-type thoracic malignant tumors located in the pleural region. Upon detection of PDPN positivity on the tumor cell membrane, the slide was directly subjected to FE-SEM analysis using the NanoSuit-CLEM method. This process involved the removal of the coverslip, DAB enhancement with osmium, and coating of the immunostained tissue with SSE solution. The presence of well-developed microvilli at immunohistochemical PDPN-rich regions confirmed the mesothelial lineage, thereby leading to a diagnosis of EMPM. Conversely, the absence of these structures suggested the presence of NSCLCs. In patients with negative PDPN immunohistochemistry results, conventional immunohistochemical panels, as recommended by established guidelines [9], should be used. The NanoSuit-CLEM method facilitates rapid FE-SEM analysis of the same immunohistochemical slide and allows reapplication of the cover glass post-analysis for archival purposes. Therefore, this approach offers significant advantages over the current multi-antibody immunohistochemistry systems.

## 4. Discussion

In this study, the immunohistochemical analysis of PDPN expression in 11 cases of EMPM, 100 cases of LAC, and 23 cases of LSCC revealed significant differences in PDPN positivity among these tumor types. PDPN positivity in the tumor cell membranes was observed in 100%, 2%, and 43.5% of EMPM, LAC, and LSCC cases, respectively. Subsequent FE-SEM observations of PDPN-positive sites using the NanoSuit-CLEM method demonstrated densely packed, elongated microvilli in EMPM, whereas such structures were absent in NSCLCs. In addition, analysis of thicker sections revealed extensive microvilli coverage in the EMPM. These findings suggest that PDPN immunohistochemistry coupled with FE-SEM using the NanoSuit-CLEM method provides a practical approach for distinguishing EMPM from NSCLCs. This study is the first to demonstrate the presence of well-developed microvilli at membranous PDPN-stained sites in EMPM of FFPE sections and establish their utility in the pathological differential diagnosis of thoracic malignant tumors. Furthermore, it serves as a compelling example of how previously recognized ultrastructural findings, which were historically difficult to integrate into routine diagnostics owing to the labor-intensive nature of traditional techniques, can now be readily applied in contemporary pathological practice owing to recent methodological advancements.

Mesotheliomas, including EMPM, are rare tumors that present significant diagnostic challenges. The primary differential diagnosis for EMPM often includes NSCLCs, and diagnostic workflows typically rely on panels of mesothelial and epithelial immunohistochemical markers to establish the mesothelial lineage [9]. The most widely used mesothelial markers include calretinin, PDPN, WT1, and CK5/6, all of which demonstrate more than 80% sensitivity for EMPM [3,9,27]. However, none of these markers are entirely specific to the mesothelial origin, as they can also show positivity in subsets of carcinomas. This lack of perfect sensitivity and specificity extends to newly identified immunohistochemical markers [28,29]. Consequently, current guidelines recommend a first-line immunohistochemical panel comprising broad-spectrum cytokeratin, along with at least two mesothelial and two epithelial markers [9]. In this study, we demonstrated the utility of PDPN immunohistochemistry and subsequent FE-SEM analysis of PDPN-positive sites using the NanoSuit-CLEM method. Based on our findings, we propose a simplified diagnostic workflow in which PDPN immunohistochemistry serves as the initial diagnostic test for epithelioid-type malignant thoracic tumors located in the pleural region (Figure 6). If PDPN positivity is detected on the tumor cell membrane, the slide can be directly processed for FE-SEM analysis using the NanoSuit-CLEM method. The presence of well-developed microvilli confirms a mesothelial lineage, supporting the diagnosis of EMPM, whereas their absence suggests NSCLCs. For cases in which PDPN is negative on the initial immunohistochemical analysis, conventional immunohistochemical panels, as recommended by existing guidelines [9], should be utilized. The NanoSuit-CLEM method facilitates rapid SEM analysis of the same immunohistochemical slide, offering significant practical advantages, including streamlined diagnostic workflows and reduced reliance on extensive antibody panels.

The ultrastructural characteristics of mesothelioma are well documented, with the presence of well-developed microvilli on the cell membranes of EMPM, which serve as a distinguishing feature that is absent in LAC [30,31,32]. PDPN, a type I transmembrane glycoprotein with an extracellular domain, a transmembrane region, and a short cytoplasmic tail, is concentrated in plasma membrane extensions, such as microvilli [33]. Consequently, SEM observation of membranous PDPN-rich sites in the EMPM highlights the presence of microvilli. In contrast, PDPN expression in NSCLCs is likely independent of microvilli, as it is localized to irregular plasma membrane structures other than microvilli. Our study successfully demonstrates that the presence or absence of microvilli in PDPN-rich regions facilitates the differential diagnosis of EMPM and NSCLCs. Furthermore, although microvilli in mesotheliomas have traditionally been ultrastructurally observed in samples fixed using conventional methods (e.g., glutaraldehyde fixation) [30,31,32], their preservation in FFPE sections has not been previously established. Using the NanoSuit-CLEM method, we demonstrated that microvilli were preserved in mesothelioma cells within FFPE sections, making this approach highly valuable for the differential diagnosis of thoracic malignancies.

An approach combining the NanoSuit-CLEM method with energy-dispersive X-ray spectroscopy (EDS) was recently developed [17]. This technique enables the elemental analysis of FFPE tissue specimens, facilitates the identification of metal deposits, and contributes to advancements in medical diagnostics. For instance, lanthanum phosphate deposits in the gastrointestinal tract can be rapidly detected in patients undergoing lanthanum carbonate treatment, facilitating pathological diagnosis [19]. Additionally, this approach provided insights into gastric black spots observed in patients with a history of *Helicobacter pylori* (*H. pylori*) eradication therapy [34]. In contrast to individuals with current *H. pylori* infection who rarely exhibit black spots, those with a history of eradication therapy frequently present with these lesions. Elemental analysis of the gastric black spots using this method revealed that iron was the primary component. The applicability of the NanoSuit-CLEM combined with SEM-EDS extends beyond the gastrointestinal tract and is promising for use in other organs. For instance, exogenous substances are constantly inhaled into the lungs, and elemental analysis of FFPE lung specimens may help elucidate previously unidentified aspects of pulmonary pathology [35,36,37,38]. Moreover, this method could potentially contribute to differentiating EMPM from NSCLC using an alternative approach to our proposed workflow (Figure 6), further enhancing its diagnostic utility in pulmonary pathology.

A limitation of the present study is the relatively small number of EMPM cases (*n* = 11). Although clear differences in PDPN positivity and the ultrastructural appearance of microvilli were demonstrated in comparison with LAC and LSCC, further validation is required. A future multi-institutional study involving a larger cohort of EMPM cases and the application of FE-SEM using the NanoSuit-CLEM method would enable a concrete conclusion regarding the utility of this approach in distinguishing EMPM from NSCLCs.

Advances in electron microscopy technology have led to the development of desktop and tabletop electron microscopes, as well as high-performance instruments [39,40]. As demonstrated in this study, SEM analysis using the NanoSuit-CLEM method on conventional pathological slides, including those stained with H&E, special stains, and immunohistochemistry, offers significant potential in clinical pathology. The combination of such accessible electron microscopy technologies with the NanoSuit-CLEM method could greatly expand the use of electron microscopy in routine pathological diagnostics.

## 5. Conclusions

A combined application of PDPN immunohistochemistry and FE-SEM using the NanoSuit-CLEM method provides an effective approach for distinguishing EMPM from NSCLCs. Consistent PDPN positivity in the EMPM and the presence of densely packed elongated microvilli at PDPN-positive sites are valuable diagnostic markers. This method bridges the gap between conventional immunohistochemistry and ultrastructural pathology, enabling a more precise tumor classification.

## Data Availability

The data presented in this study are available on request from the corresponding author.

## Supplementary Materials

The following is available online at https://www.mdpi.com/article/10.3390/diagnostics15101298/s1, Figure S1: Representative immunohistochemical images of an EMPM case, Figure S2: Representative immunohistochemical image showing lymphatic vessel detection in a thoracic malignant tumor using PDPN immunohistochemistry, Figure S3: Original data, Table S1: Age and sex distribution of patients with EMPM, Table S2: Clinicopathological characteristics of patients with LAC, Table S3: Clinicopathological characteristics of patients with LSCC, Table S4: Clinicopathological factors in 100 LAC cases stratified by PDPN immunostaining status, Table S5: Clinicopathological factors in 23 LSCC cases stratified by PDPN immunostaining status.

## Abbreviations

The following abbreviations are used in this manuscript:

BSE	Backscattered electron
CLEM	Correlative light and electron microscopy
DAB	3,3′-diaminobenzidine tetrahydrochloride
EDS	Energy-dispersive X-ray spectroscopy
EMPM	Epithelioid malignant pleural mesothelioma
FE-SEM	Field emission–scanning electron microscopy
FFPE	Formalin-fixed paraffin-embedded
H&E	Hematoxylin and eosin
*H. pylori*	*Helicobacter pylor**i*
LAC	Lung adenocarcinoma
LSCC	Lung squamous cell carcinoma
PDPN	Podoplanin
SSE	Surface shield enhancer
YAG	Yttrium aluminum garnet
