# Supplementary material for: Diagnostic Utility of Podoplanin Immunohistochemistry Combined with the NanoSuit-Correlative Light and Electron Microscopy Method for Thoracic Malignant Tumors
Source: Diagnostics (Basel). 2025 May 21;15(10):1298. doi: 10.3390/diagnostics15101298 (PMC12109644; doi:10.3390/diagnostics15101298)
Supplement: Supplementary file 1 [file diagnostics-15-01298-s001.zip › diagnostics-3543505-supplementary.pdf]

Supplementary Information

Diagnostic Utility of Podoplanin Immunohistochemistry Combined with the NanoSuit-Correlative Light and Electron Microscopy Method for Thoracic Malignant Tumors

Supplementary Figure S1: Representative immunohistochemical images of an EMPM case

Supplementary Figure S2: Representative immunohistochemical image showing lymphatic vessel detection in a thoracic malignant tumor using PDPN immunohistochemistry

Supplementary Figure S3: Original data

Supplementary Table S1: Age and sex distribution of patients with EMPM

Supplementary Table S2: Clinicopathological characteristics of patients with LAC

Supplementary Table S3: Clinicopathological characteristics of patients with LSCC

Supplementary Table S4: Clinicopathological factors in 100 LAC cases stratified by PDPN immunostaining status

Supplementary Table S5: Clinicopathological factors in 23 LSCC cases stratified by PDPN immunostaining status

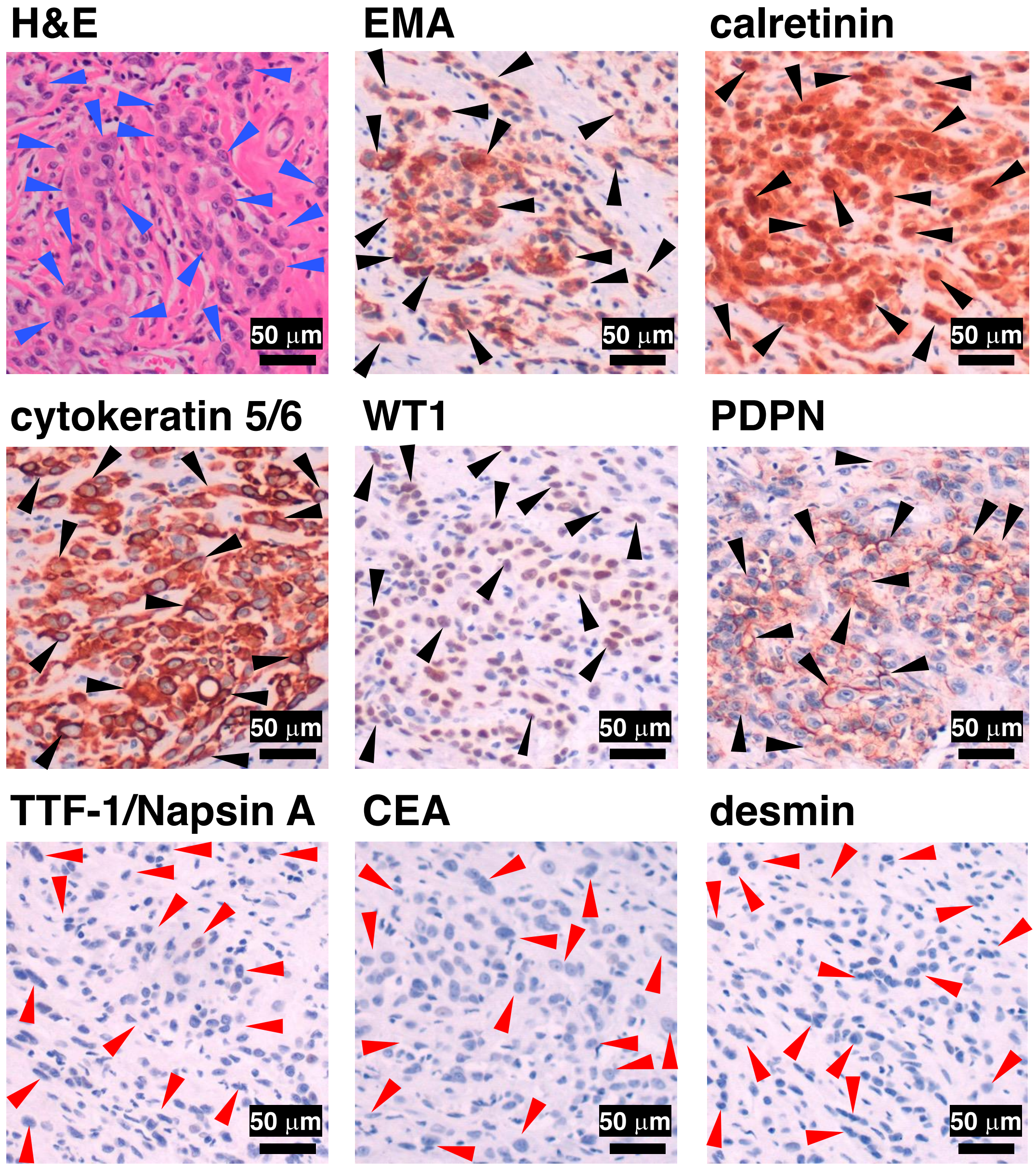

Supplementary Figure S1: Representative immunohistochemical images of an EMPM case. The tumor cells in this case demonstrated diffuse positivity for the epithelial/mesothelial marker EMA. In addition, they exhibited positive immunoreactivity for calretinin, cytokeratin 5/6, WT1, and PDPN, all of which are established markers for EMPM, while showing negative staining for TTF-1, Napsin A, and CEA, which are markers characteristic of LAC. The tumor cells were also negative for the muscle marker desmin. The subcellular localization of the immunopositive signals was as follows: EMA, cytoplasmic; calretinin, nuclear and cytoplasmic; cytokeratin 5/6, cytoplasmic; WT1, nuclear; and PDPN,

membranous. These immunohistochemical findings are consistent with the diagnosis of EMPM. The primary antibodies used were as follows: anti-EMA monoclonal antibody (mAb) [clone E29; heat-induced epitope retrieval (HIER) using citrate buffer (pH 6.0); dilution 1:100; Cell Marque, Rocklin, CA, USA], anti-calretinin mAb [clone SP13; HIER using Tris-EDTA buffer (pH 9.0); dilution 1:100; Nichirei Biosciences, Tokyo, Japan], anti-WT1 mAb [clone WT49; HIER with Tris-EDTA buffer (pH 9.0); dilution 1:50; Leica Biosystems, Buffalo Grove, IL, USA], anti-PDPN mAb (clone D2-40; no antigen retrieval; dilution 1:200; DAKO, Carpinteria, CA, USA), anti-cytokeratin 5/6 mAb [clone D5/16 B4; HIER with Tris-EDTA buffer (pH 9.0); dilution 1:50; DAKO], anti-TTF-1 mAb (clone SPT24; HIER with Tris-EDTA buffer (pH 9.0); dilution 1:100; Leica Biosystems), anti-Napsin A mAb [clone IP64; HIER with Tris-EDTA buffer (pH 9.0); dilution 1:800; Leica Biosystems], anti-CEA mAb [clone Ⅱ-7; HIER with citrate buffer (pH 6.0); dilution 1:200; DAKO], anti-desmin mAb [clone DE-R-11; HIER with Tris-EDTA buffer (pH 9.0); dilution 1:200; Leica Biosystems]. In the images, black arrowheads indicate PDPN-positive tumor cells; blue arrowheads indicate tumor growth architecture in the H&E-stained section; and red arrowheads indicate tumor cells that are negative for the respective immunohistochemical markers.

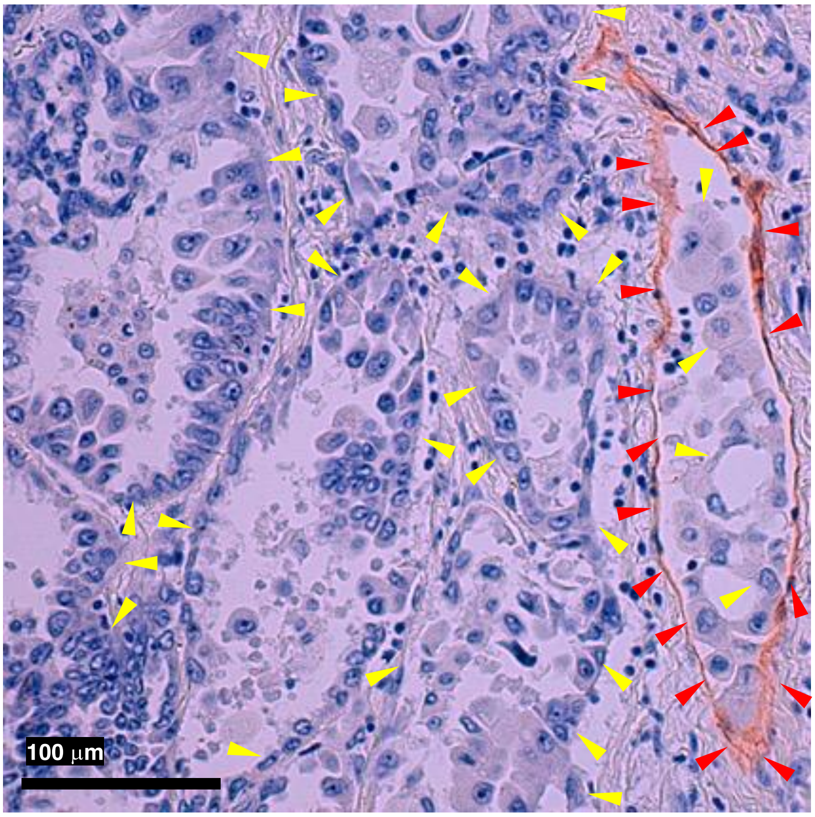

Supplementary Figure S2: Representative immunohistochemical image showing lymphatic vessel detection in a thoracic malignant tumor using PDPN immunohistochemistry. This analysis was conducted in a case of LAC exhibiting lymphatic invasion. Red arrowheads indicate PDPN-positive lymphatic vessels, and yellow arrowheads indicate tumor cells, some of which are observed within the lumen of the lymphatic vessel.

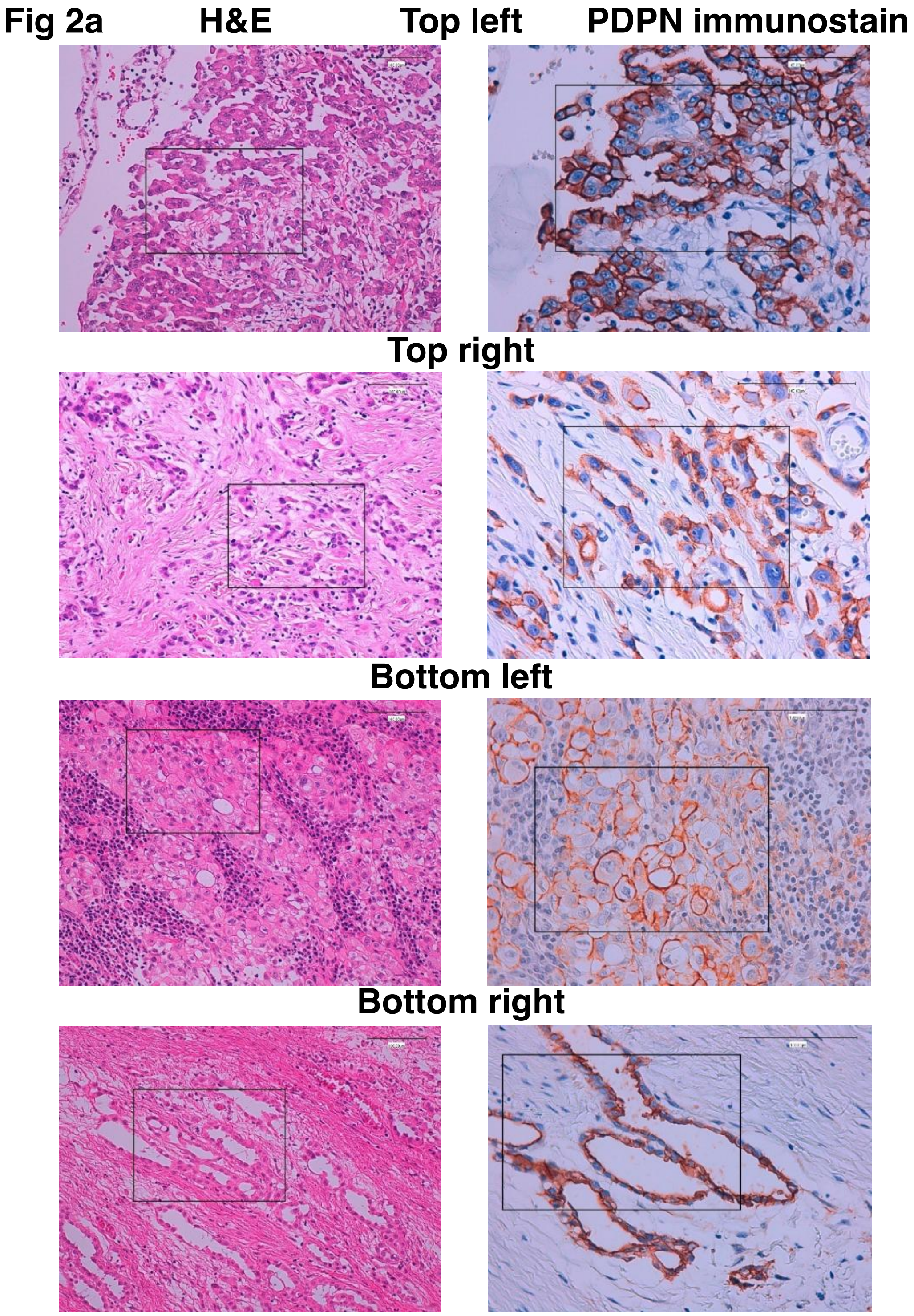

Supplementary Figure S3: Original data. Cropped regions used in the main figures are indicated by black rectangles on the original images.

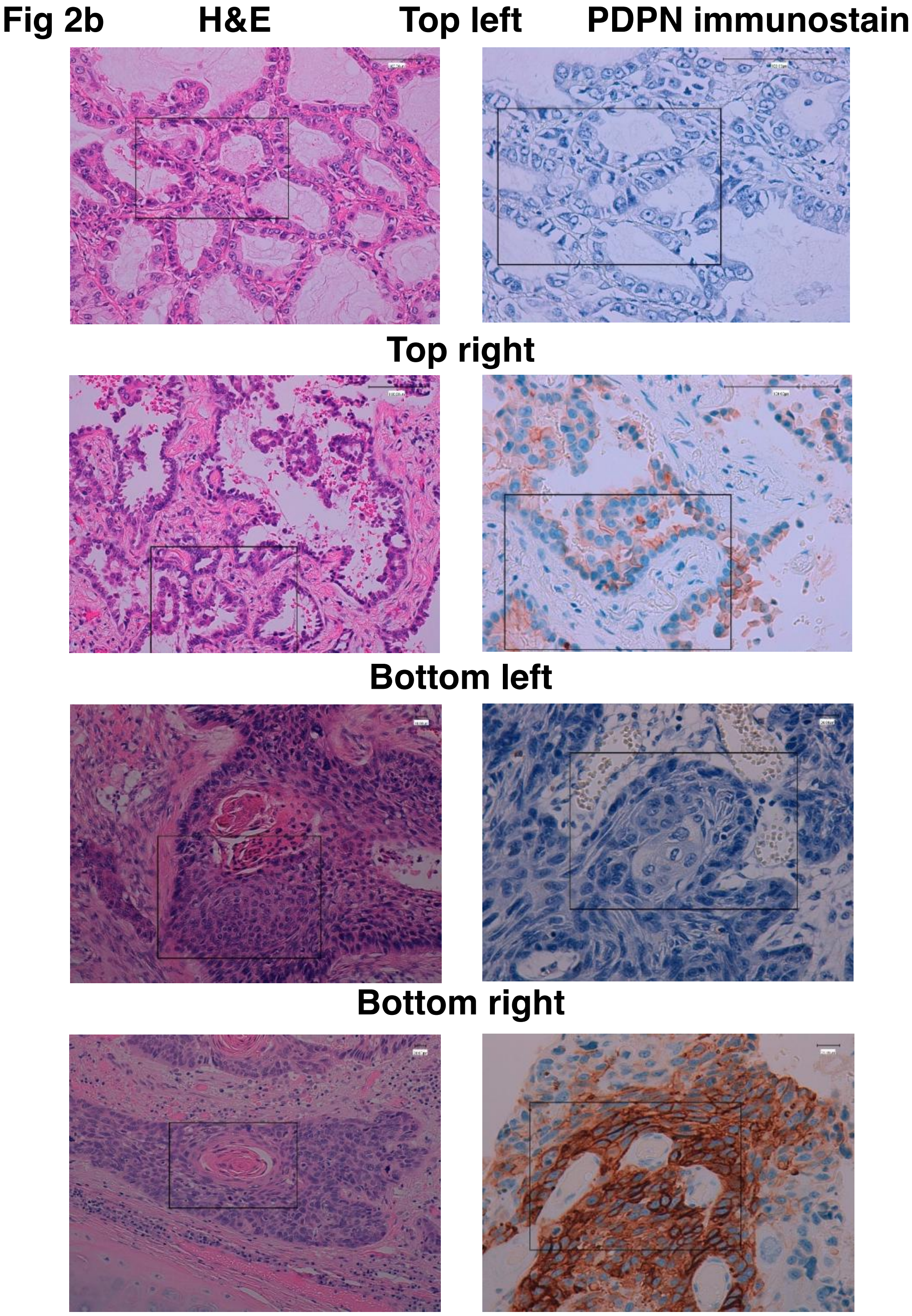

Supplementary Figure S3: Original data (Continued). Cropped regions used in the main figures are indicated by black rectangles on the original images.

Fig 3a

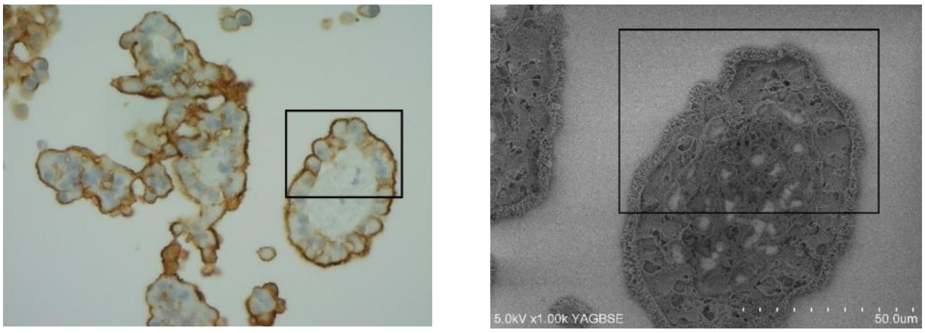

Fig 3b

Case 1

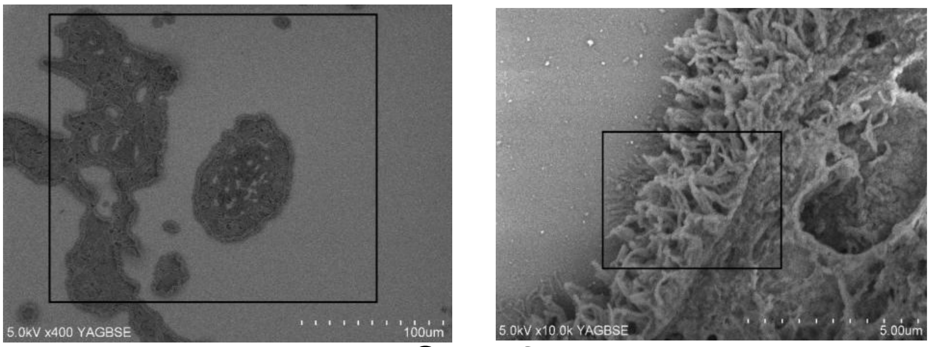

Case 2

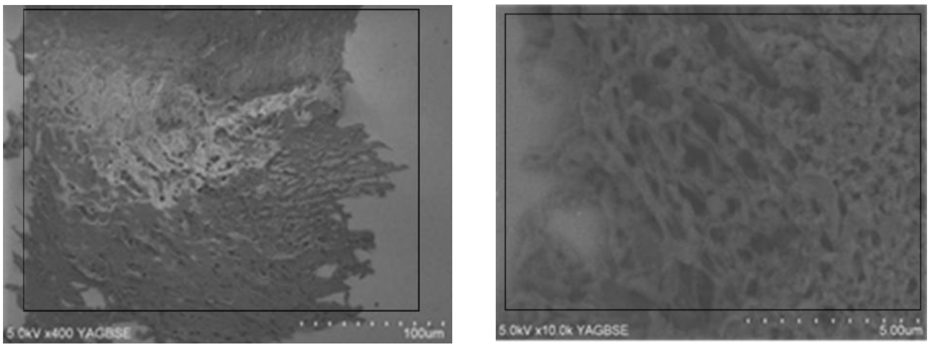

Case 3

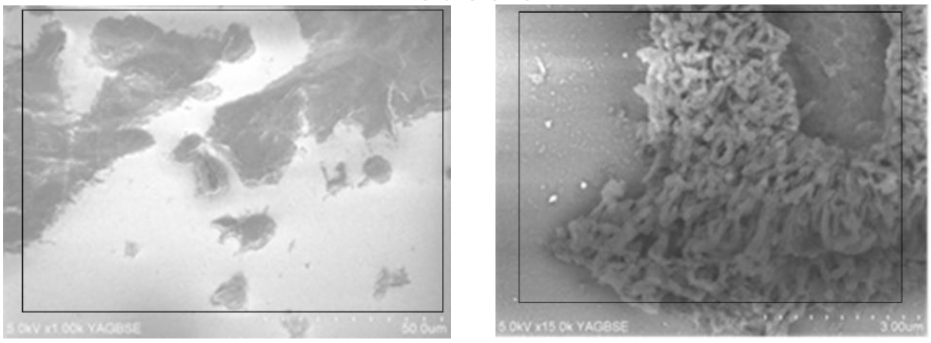

Supplementary Figure S3: Original data (Continued). Cropped regions used in the main figures are indicated by black rectangles on the original images.

Fig 4

LAC **LSCC**

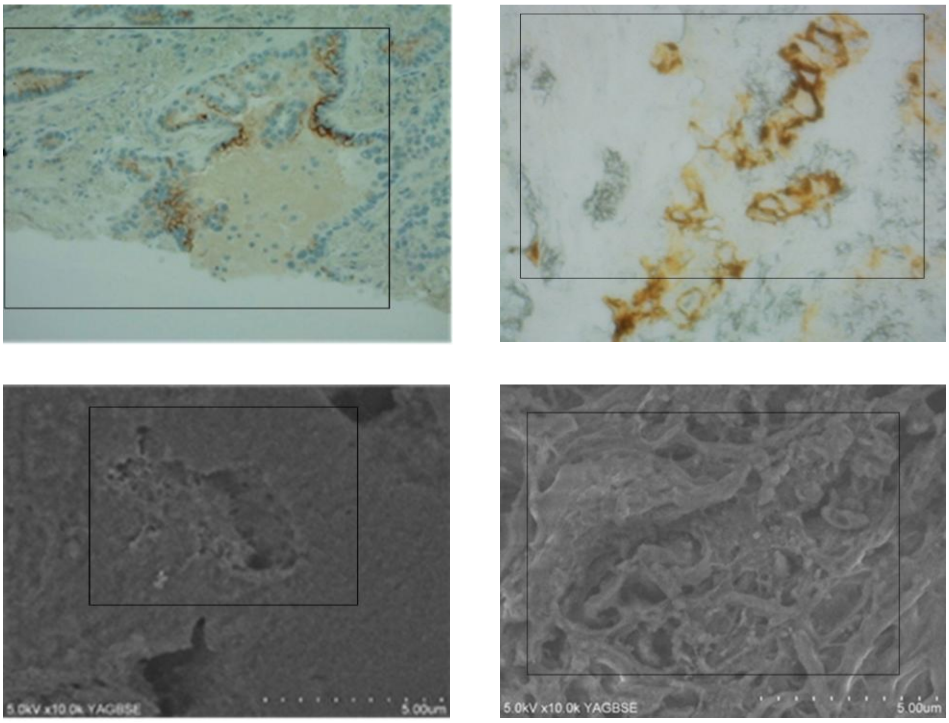

Fig 5

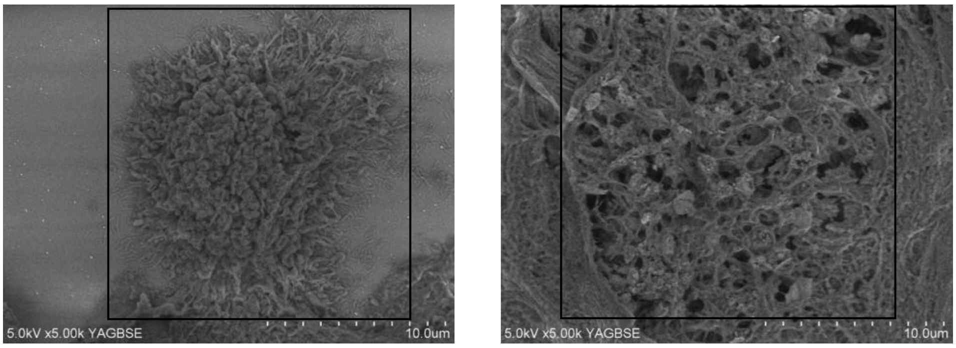

Supplementary Figure S3: Original data (Continued). Cropped regions used in the main figures are indicated by black rectangles on the original images.

Supplementary Table S1: Age and sex distribution of patients with EMPM.

Case ID	Age	Sex	Type
No. 1	76	M	Epithelioid pleural mesothelioma
No. 2	61	F	Epithelioid pleural mesothelioma
No. 3	61	M	Epithelioid pleural mesothelioma
No. 4	74	M	Epithelioid pleural mesothelioma
No. 5	64	M	Epithelioid pleural mesothelioma
No. 6	50	M	Epithelioid pleural mesothelioma
No. 7	59	M	Epithelioid pleural mesothelioma
No. 8	56	M	Epithelioid pleural mesothelioma
No. 9	62	M	Epithelioid pleural mesothelioma
No. 10	47	M	Epithelioid pleural mesothelioma
No. 11	41	F	Epithelioid pleural mesothelioma

M, male; F, female.

Supplementary Table S2: Clinicopathological characteristics of patients with LAC.

Case ID	Age	Sex	Type	pT	pN	Ly[a]	V[b]	pm[c]	pl[d]
No. 1	80	F	INMA	1	0	1	1	0	0
No. 2	57	F	INMA	1	0	0	0	0	0
No. 3	68	F	INMA	1	0	0	0	0	0
No. 4	76	M	INMA	1	0	0	0	0	0
No. 5	71	M	INMA	2	2	1	1	0	0
No. 6	81	M	INMA	2	0	0	1	0	0
No. 7	80	F	INMA	1	0	0	0	0	0
No. 8	72	M	INMA	2	0	1	1	0	1
No. 9	69	F	IMA	1	NE	0	0	0	0
No. 10	67	M	INMA	1	0	1	1	0	0
No. 11	77	M	IMA	3	0	0	1	0	1
No. 12	68	F	INMA	1	0	0	0	0	0
No. 13	74	F	INMA	1	0	0	0	0	0
No. 14	74	M	INMA	1	0	0	0	0	0
No. 15	58	F	INMA	2	2	1	1	0	1
No. 16	64	M	INMA	2	NE	1	1	0	2
No. 17	77	M	INMA	1	0	0	1	0	0
No. 18	79	F	INMA	2	NE	1	1	0	1
No. 19	67	M	INMA	2	1	1	1	0	1
No. 20	69	F	INMA	1	0	0	1	0	0
No. 21	74	M	INMA	1	2	1	0	0	0
No. 22	63	F	INMA	1	2	1	1	0	0
No. 23	78	F	INMA	1	0	0	0	0	0
No. 24	81	F	INMA	2	0	0	0	0	0
No. 25	72	F	IMA	1	0	0	0	0	0
No. 26	71	M	INMA	2	2	1	1	0	3
No. 27	73	F	INMA	1	0	0	1	0	0
No. 28	70	F	INMA	1	0	0	1	0	0
No. 29	66	M	INMA	2	0	0	1	0	3
No. 30	74	M	INMA	1	0	0	0	0	0
No. 31	69	F	INMA	1	0	0	0	0	0

Supplementary Table S2: [Continued]

Case ID	Age	Sex	Type	pT	pN	Ly[a]	V[b]	pm[c]	pl[d]
No. 32	70	F	INMA	2	2	1	1	0	2
No. 33	73	F	INMA	1	0	0	1	0	0
No. 34	53	M	INMA	2	0	0	1	0	0
No. 35	61	F	INMA	1	0	0	1	0	0
No. 36	68	F	INMA	1	0	0	1	0	0
No. 37	65	M	INMA	2	0	0	1	0	0
No. 38	72	M	IMA	1	0	0	0	0	0
No. 39	72	M	INMA	2	NE	1	1	0	1
No. 40	72	M	INMA	2	0	1	1	0	2
No. 41	59	M	INMA	1	0	0	0	0	0
No. 42	66	F	INMA	1	0	1	1	0	0
No. 43	76	M	IMA	2	0	0	0	0	0
No. 44	81	M	INMA	2	0	1	1	0	0
No. 45	64	M	INMA	1	0	0	1	0	0
No. 46	82	F	INMA	2	1	1	1	0	1
No. 47	75	F	INMA	2	2	1	1	0	1
No. 48	65	F	INMA	1	0	0	0	0	0
No. 49	85	M	INMA	2	NE	1	1	0	2
No. 50	57	M	INMA	2	0	0	1	0	1
No. 51	77	M	INMA	1	0	0	1	0	0
No. 52	58	M	INMA	2	0	0	1	0	0
No. 53	73	M	INMA	1	0	0	1	0	0
No. 54	65	F	INMA	1	0	0	0	0	0
No. 55	67	F	INMA	1	NE	0	1	0	0
No. 56	58	M	INMA	1	0	1	1	0	0
No. 57	75	F	INMA	1	0	0	0	0	0
No. 58	85	M	INMA	1	1	1	1	0	0
No. 59	61	M	INMA	2	0	1	1	0	2
No. 60	70	M	INMA	1	0	0	0	0	0
No. 61	68	F	INMA	1	NE	0	1	0	0
No. 62	60	M	INMA	2	1	1	1	0	0

Supplementary Table S2: [Continued]

Case ID	Age	Sex	Type	pT	pN	Ly[a]	V[b]	pm[c]	pl[d]
No. 63	75	F	INMA	1	0	0	1	0	0
No. 64	72	F	INMA	2	0	0	0	0	0
No. 65	73	M	INMA	2	0	1	1	0	2
No. 66	70	M	INMA	4	0	1	1	0	1
No. 67	68	F	INMA	1	0	0	1	0	0
No. 68	64	M	INMA	1	0	0	1	0	0
No. 69	73	M	INMA	4	2	1	1	0	1
No. 70	78	M	INMA	1	0	1	1	0	0
No. 71	78	M	INMA	2	0	0	1	0	3
No. 72	69	F	INMA	1	1	0	0	0	0
No. 73	69	F	INMA	2	NE	1	1	0	2
No. 74	83	M	INMA	2	0	1	1	0	0
No. 75	63	M	INMA	2	1	1	1	0	1
No. 76	67	M	INMA	2	0	0	1	0	1
No. 77	47	F	INMA	1	0	0	0	0	0
No. 78	69	M	INMA	2	0	1	1	0	0
No. 79	79	F	INMA	1	0	0	1	0	0
No. 80	56	M	INMA	2	0	1	1	0	1
No. 81	69	M	INMA	1	0	0	0	0	0
No. 82	72	F	INMA	2	0	0	0	0	0
No. 83	67	M	INMA	1	0	0	0	0	0
No. 84	74	M	INMA	1	0	1	1	0	0
No. 85	75	M	INMA	1	0	0	0	0	0
No. 86	77	M	INMA	1	0	0	0	0	0
No. 87	78	M	INMA	1	0	0	0	0	0
No. 88	70	F	INMA	1	0	0	0	0	0
No. 89	61	F	INMA	2	0	1	1	0	0
No. 90	73	F	INMA	1	0	0	1	0	0
No. 91	76	F	INMA	1	0	0	0	0	0
No. 92	77	F	INMA	1	0	0	0	0	0
No. 93	50	F	INMA	1	0	0	0	0	0

Supplementary Table S2: [Continued]

Case ID	Age	Sex	Type	pT	pN	Ly[a]	V[b]	pm[c]	pl[d]
No. 94	77	M	INMA	1	0	1	1	0	0
No. 95	67	F	INMA	1	2	1	1	0	0
No. 96	65	M	INMA	1	0	0	0	0	0
No. 97	65	F	INMA	2	0	0	1	0	0
No. 98	81	F	INMA	1	0	0	1	0	0
No. 99	74	M	INMA	1	NE	0	0	0	0
No. 100	83	M	INMA	2	0	1	0	0	1

M, male; F, female; INMA, invasive non-mucinous adenocarcinoma; IMA, invasive mucinous adenocarcinoma; NE, not examined; Ly, lymphatic invasion; V, vascular invasion; pm, intrapulmonary metastasis; pl, pleural invasion.

Ly[a]: Lymphatic invasion was assessed according to the Japanese Classification of Lung Cancer, with Ly0 indicating absence and Ly1 indicating presence of lymphatic invasion.

V[b]: Vascular invasion was assessed according to the Japanese Classification of Lung Cancer, with V0 indicating absence and V1 indicating presence of vascular invasion.

pm[c]: Intrapulmonary metastasis was assessed according to the Japanese Classification of Lung Cancer, with pm0 indicating absence and pm1 indicating presence of intrapulmonary metastasis.

pl[d]: Pleural invasion was assessed according to the Japanese Classification of Lung Cancer, with pl1 indicating invasion beyond the elastic layer, pl2 indicating invasion to the surface of the visceral pleura, and pl3 indicating invasion beyond the parietal pleura into the chest wall, diaphragm, mediastinal organs, or into an adjacent lobe across the interlobar fissure.

Supplementary Table S3: Clinicopathological characteristics of patients with LSCC.

Case ID	Age	Sex	pT	pN	Ly[a]	V[b]	pm[c]	pl[d]
No. 1	63	M	2	0	0	0	0	0
No. 2	71	M	2	1	1	1	0	2
No. 3	72	F	1	0	0	1	0	0
No. 4	73	M	1	0	0	1	0	0
No. 5	75	M	3	2	1	1	0	3
No. 6	66	M	2	2	1	1	0	1
No. 7	73	M	1	0	0	1	0	0
No. 8	70	M	1	0	0	0	0	0
No. 9	74	M	2	0	0	1	0	3
No. 10	67	M	2	0	0	1	0	0
No. 11	76	M	3	0	1	1	0	3
No. 12	75	M	3	0	1	1	0	3
No. 13	76	M	2	0	1	1	0	0
No. 14	67	M	2	0	0	1	0	0
No. 15	71	M	2	0	1	1	0	1
No. 16	72	M	2	0	0	1	0	1
No. 17	58	M	3	0	1	1	0	3
No. 18	81	M	1	0	1	1	0	0
No. 19	78	M	1	0	0	1	0	0
No. 20	74	F	2	NE	0	0	0	0
No. 21	68	M	2	0	0	1	0	0
No. 22	80	M	3	0	0	1	0	0
No. 23	75	F	2	0	0	1	0	0

M, male; F, female; NE, not examined; Ly, lymphatic invasion; V, vascular invasion; pm, intrapulmonary metastasis; pl, pleural invasion.

Ly[a]: Lymphatic invasion was assessed according to the Japanese Classification of Lung Cancer, with Ly0 indicating absence and Ly1 indicating presence of lymphatic invasion.

V[b]: Vascular invasion was assessed according to the Japanese Classification of Lung Cancer, with V0 indicating absence and V1 indicating presence of vascular invasion.

pm[c]: Intrapulmonary metastasis was assessed according to the Japanese Classification of Lung Cancer, with pm0 indicating absence and pm1 indicating presence of intrapulmonary metastasis.
pl[d]: Pleural invasion was assessed according to the Japanese Classification of Lung Cancer, with pl1 indicating invasion beyond the elastic layer, pl2 indicating invasion to the surface of the visceral pleura, and pl3 indicating invasion beyond the parietal pleura into the chest wall, diaphragm, mediastinal organs, or into an adjacent lobe across the interlobar fissure.

Supplementary Table S4: Clinicopathological factors in 100 LAC cases stratified by PDPN immunostaining status

**Factor**	**No. of cases**	**PDPN immunostaining status**		***P*-value**†
		**Negative (n = 98)**	**Positive (n = 2)**	
Age				
<70	44	43 (97.7%)	1 (2.3%)	1.0000
≥70	56	55 (98.2%)	1 (1.8%)	
Sex				
Female	46	44 (95.7%)	2 (4.4%)	0.2091
Male	54	54 (100.0%)	0 (0%)	
Histology				
IMA	5	5 (100.0%)	0 (0%)	1.0000
INMA	95	93 (97.9%)	2 (2.1%)	
pT				
pT1	60	58 (96.7%)	2 (3.3%)	0.5152
pT2-pT4	40	40 (100.0%)	0 (0%)	
pN				
pN0	76	74 (97.4%)	2 (2.7%)	1.0000
pN1-pN3	15	15 (100.0%)	0 (0%)	
Lymphatic invasion				
Absence	63	62 (98.4%)	1 (1.6%)	1.0000
Presence	37	36 (97.3%)	1 (2.7%)	
Vascular invasion				
Absence	37	37 (100.0%)	0 (0%)	0.5291
Presence	63	61 (96.8%)	2 (3.2%)	
Intrapulmonary metastasis				
Absence	100	98 (98.0%)	2 (2.0%)	NA
Presence	0	0	0	
Pleural invasion				
Absence	75	73 (97.3%)	2 (2.7%)	1.0000
Presence	25	25 (100.0%)	0 (0%)	

IMA, invasive mucinous adenocarcinoma; INMA, invasive non-mucinous adenocarcinoma; NA, not applicable. †Fisher's exact test.

Supplementary Table S5: Clinicopathological factors in 23 LSCC cases stratified by PDPN immunostaining status

Factor	No. of cases	PDPN immunostaining status		*P*-value†
		Negative (n = 13)	Positive (n = 10)	
Age				
<70	6	5 (83.3%)	1 (16.7%)	0.1790
≥70	17	8 (47.1%)	9 (52.9%)	
Sex				
Female	3	1 (33.3%)	2 (66.7%)	0.5596
Male	20	12 (60.0%)	8 (40.0%)	
pT				
pT1	6	2 (33.3%)	4 (66.7%)	0.3413
pT2-pT4	17	11 (64.7%)	6 (35.3%)	
pN				
pN0	19	11 (57.9%)	8 (42.1%)	1.0000
pN1-pN3	3	2 (66.7%)	1 (33.3%)	
Lymphatic invasion				
Absence	14	6 (42.9%)	8 (57.1%)	0.1968
Presence	9	7 (77.8%)	2 (22.2%)	
Vascular invasion				
Absence	3	0 (0%)	3 (100.0%)	0.0678
Presence	20	13 (65.0%)	7 (35.0%)	
Intrapulmonary metastasis				
Absence	23	13 (56.5%)	10 (43.5%)	NA
Presence	0	0	0	
Pleural invasion				
Absence	14	8 (57.1%)	6 (42.9%)	1.0000
Presence	9	5 (55.6%)	4 (44.4%)	

NA, not applicable. †Fisher's exact test.
